# Colonization of Honey Bee Digestive Tracts by Environmental Yeast *Lachancea thermotolerans* Is Naturally Occurring, Temperature Dependent, and Impacts the Microbiome of Newly Emerged Bees

**DOI:** 10.1128/spectrum.05194-22

**Published:** 2023-02-15

**Authors:** Helen V. Kogan, Annabelle B. Elikan, Kimberly F. Glaser, Jenna M. Bergmann, Laure M. Raymond, Sofia R. Prado-Irwin, Jonathan W. Snow

**Affiliations:** a Biology Department, Barnard College, New York, New York, USA; Agroscope

**Keywords:** fungus, yeast, honey bee, colonization, digestive tract, temperature, microbiome

## Abstract

Honey bees are critical pollinators in both agricultural and ecological settings. Recent declines in honey bee colonies in the United States have put increased strain on agricultural pollination. Although there are many environmental stressors implicated in honey bee disease, there has been intensifying focus on the role of microbial attacks on honey bee health. Despite the long-standing appreciation for the association of fungi of various groups with honey bees and their broader environment, the effects of these interactions on honey bee health are incompletely understood. Here, we report the discovery of colonization of the honey bee digestive tract by the environmental yeast Lachancea thermotolerans. Experimental colonization of honey bee digestive tracts by L. thermotolerans revealed that this yeast species maintains high levels in the honey bee midgut only at temperatures below the typical colony temperature. In newly eclosed bees, *L. thermotolerans* colonization alters the microbiome, suggesting that environmental yeasts can impact its composition. Future studies should be undertaken to better understand the role of *L. thermotolerans* and other environmental yeasts in honey bee health.

**IMPORTANCE** Although many fungal species are found in association with honey bees and their broader environment, the effects of these interactions on honey bee health are largely unknown. Here, we report the discovery that a yeast commonly found in the environment can be found at high levels in honey bee digestive tracts. Experimentally feeding this yeast to honey bees showed that the yeast’s ability to maintain high levels in the digestive tract is influenced by temperature and can lead to alterations of the microbiome in young bees. These studies provide a foundation for future studies to better understand the role of environmental yeasts in honey bee health.

## INTRODUCTION

The Western honey bee, Apis mellifera, provides critical pollination services of paramount importance to humans in agricultural settings (1). Honey bee colonies in the United States have suffered from an increased rate of die-off in recent years that likely stems from a complex set of interacting stresses that remain poorly described. Key stresses thought to be involved include nutritional stress due to the loss of appropriate forage, chemical poisoning from pesticides, changes to normal living conditions brought about by large-scale beekeeping practices, and infection by pathogenic microbes (2).

Among the environmental stressors implicated in honey bee disease, there has been intensifying focus on the role of microbial infection on honey bee health (3). Starting with the advent of colony collapse disorder in 2006, the search for novel microbial pathogens that might be to blame for increased honey bee colony deaths has been extensive. As microbial diseases affecting larvae are well appreciated, particular emphasis has been placed on microbes found in adult bees, with those in the digestive tract receiving particular interest. This effort has produced truly remarkable advances in our understanding of the types, levels, and community architectures of pathogenic and nonpathogenic microbes associated with honey bees and their hives (reviewed in reference 3). A number of fungal species are known to cause disease in larvae (4), including Ascosphaera apis, which causes chalkbrood (5), and Aspergillus species, which cause stonebrood (6). In addition, Aspergillus species have been shown to cause disease in adult bees (7). However, other than these species and the well-known microsporidian species Vairimorpha (Nosema) apis and Vairimorpha (Nosema) ceranae, the effects of novel fungi on honey bee health have been relatively understudied in this endeavor. In the first metagenomic analysis of adult honey bee-associated microbes, three additional classes of fungi were identified: the *Entomophthorales*, *Saccharomycotina* (yeasts), and *Mucoromycotina* (molds) (8). The association of fungi of these three groups with honey bees and their broader environment is not new (9–18). However, the role that yeasts play in honey bee health is only partially understood. They can be found in floral nectaries (19, 20) and on pollen (13) and have been observed in the hive environment. Some yeast species have also been found to be common inhabitants of the digestive tract of adult honey bees in the extensive and pioneering work of Gilliam. Evidence from this body of work suggests that their prevalence and abundance are affected by stress (14, 15, 17, 18), and it is possible that such yeast species represent opportunistic pathogens of honey bees. Newer studies have continued to leverage sequencing methods to identify novel fungi, in particular yeasts, in bees and their environment (21–24) and to explore the role of these species in bee health and disease (25–27). However, additional studies using isolates to functionally characterize the interactions between bees and these fungal species will be critical.

Here, we describe the isolation and characterization of a yeast species in the honey bee digestive tract. During a routine inspection of colonies for *Vairimorpha* infection in the midgut, we discovered the presence of yeast-like microbial cells that were subsequently identified as Lachancea thermotolerans. In a limited apiary-wide survey of colonies in our apiary, we observed the presence of this yeast only in early fall. Oral infection experiments suggest that this yeast maintains high levels in the honey bee midgut only at temperatures below the typical colony temperature. The observed impacts of temperature on the L. thermotolerans levels in the honey bee midgut are potentially mediated through effects on the microbe, the host, or both. However, we show that *L. thermotolerans* prefers a lower growth temperature in culture, suggesting that the temperature effect is mediated at least in part by yeast-intrinsic factors. We also observe that *L. thermotolerans* inoculation causes microbiome perturbations in newly eclosed bees but not older bees, suggesting an interplay between this environmental yeast and the bacterial constituents of the microbiome.

## RESULTS

### Isolation of a yeast species from the digestive tracts of honey bees.

**FIG 1:**
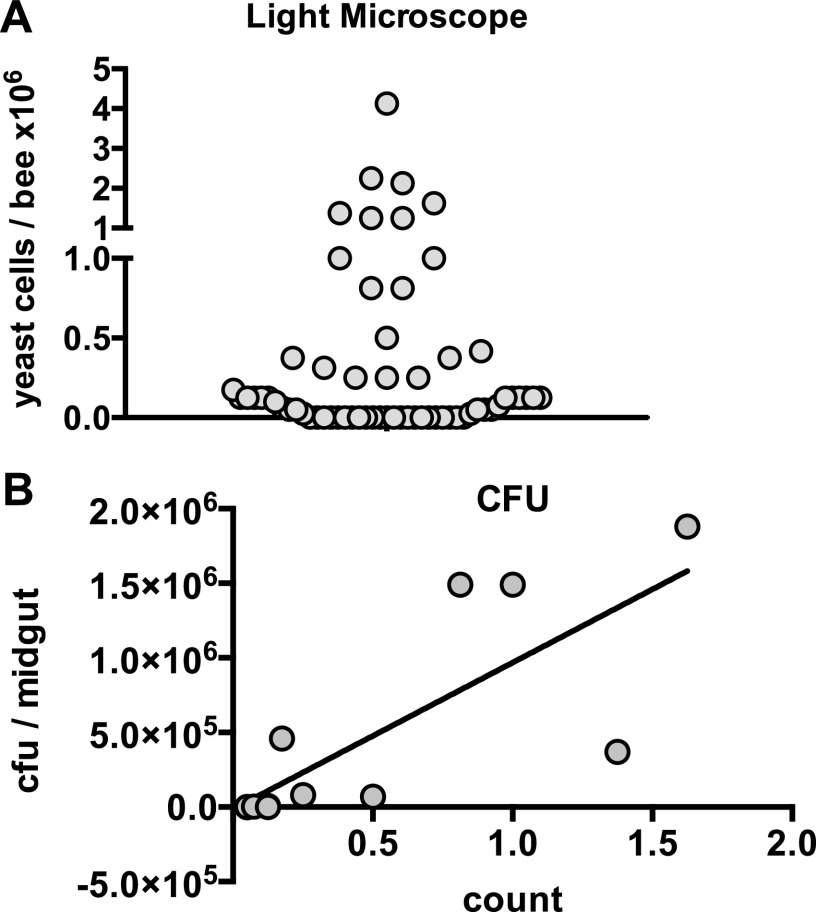
Yeast cells are observed in honey bee midguts. Yeast cell levels in honey bee midguts were determined by cell counts using light microscopy (A) or by plating onto YPD plates containing streptomycin (B).

While monitoring multiple colonies for Vairimorpha ceranae levels as part of ongoing studies in 2015, we noted the presence of unique cells in the midguts of bees from a specific colony in early October that resembled yeast cells. Combination of three independent sampling events for this colony showed that it had a 63.2% prevalence of infection (Fig. 1A). Similar cells were observed in two other colonies. We also measured CFU by plating the cells onto yeast extract-peptone-dextrose (YPD) with streptomycin to prevent bacterial growth and incubating the plates overnight at 35°C (honey bee colony temperature) under aerobic conditions. Microscopic counts correlated well with CFU (Fig. 1B), and microscopic examination of cells making up the colony revealed cells with a morphology similar to that of the cells described above.

### Chitin-binding agents render yeast cell-like structures visible by fluorescence microscopy.

**FIG 2:**
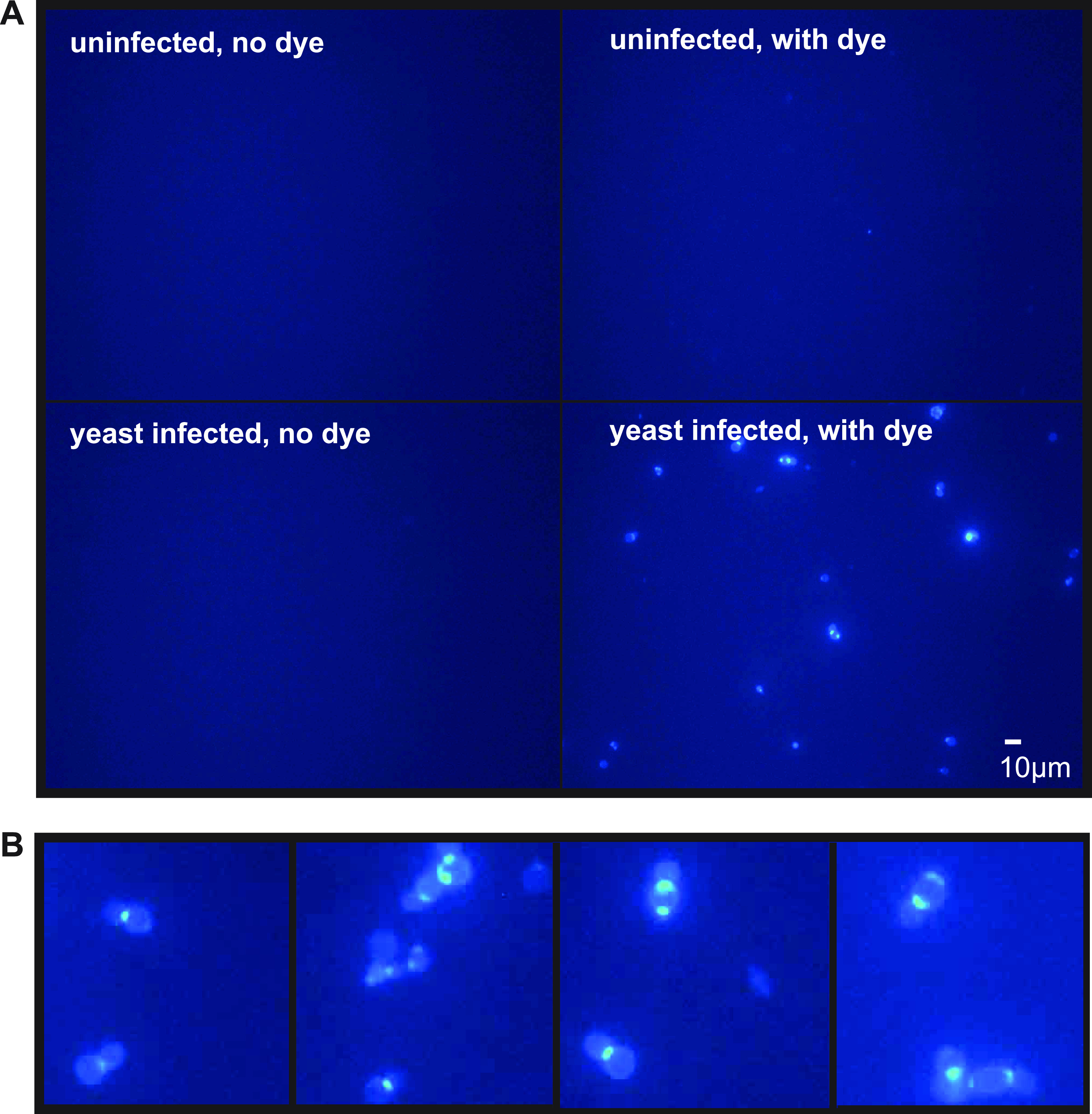
Chitin-binding fluorescent brightener 28 allows visualization of yeast cells. Midgut preparations from an uninfected bee and an infected bee from an infected colony with or without fluorescent brightener 28 were visualized using UV with a 40× objective.

To demonstrate that the cells were of fungal origin, we used the chitin-binding agent fluorescent brightener 28 (FB28), which is also known as calcofluor white M2R. Using fluorescence microscopy, we could see that the dye was specific for structures resembling yeast cells and that no signal was observed in the absence of the dye (Fig. 2A) in the midgut of a bee with high yeast cell counts by light microscopy, and no similar structures were observed in the midgut of a bee with no yeast cells. In addition, clear yeast-like forms were observed, including budding cells with chitin-rich neck areas (Fig. 2B). We previously showed that this dye binds to V. ceranae spore walls (28, 29), and we sought to determine whether FB28 binding can differentiate between the two types of fungi. At the exposure times used to visualize V. ceranae, the yeast cells were too faint to observe, suggesting that the amount of chitin per *Vairimorpha* spore is significantly larger than that on the yeast cells. In addition, V. ceranae spores were easily differentiated based on morphology (see Fig. S1 in the supplemental material).

### Yeast species identification, isolation, and characterization.

Sequence analysis of RNA obtained from a midgut that was visually confirmed to contain the yeast was performed using fungus-specific primers ITS1F and ITS2, and the yeast species was identified as *Lachancea thermotolerans* (Fig. S2). We isolated the yeast from an aliquot of the infected midgut described above by plating cells onto YPD and incubating the plate overnight at 35°C (honey bee colony temperature) under aerobic conditions. Sequencing of the RNA extracted from a liquid culture inoculated with a single colony of the plated yeast using the fungus-specific primers ITS1F and ITS2, or NL1 and NL4, confirmed that the yeast species was *L. thermotolerans*. The species had 99% identity with isolate KT419 of *L. thermotolerans* for the internal transcribed spacer 1 (ITS1) sequence (GenBank accession number KY558362.1) and 100% sequence identity with the CBS 6340 strain for the D1-D2 region (GenBank accession number XR_002432231.1).

### Temporal pattern of *L. thermotolerans* over a season.

We developed a quantitative PCR (qPCR) assay to determine the levels of the *L. thermotolerans* 26S ribosomal DNA (rDNA) (30) relative to the levels of the honey bee nuclear ATPase 5a gene (*atp5a*) in the midguts of infected and uninfected individuals. We then used this assay to quantify *L. thermotolerans* and V. ceranae over a single season (21 February to 21 November 2017) in our apiary, which contained between 5 and 8 colonies (numbers of colony samples at each time point can be found in Fig. S3). The intensity of V. ceranae infection was variable among colonies, and at least one colony was infected at each time point, but the apiary mean reached a peak in July (Fig. S3). Fumagillin was administered starting in September, and infection levels decreased, likely in response to treatment and the arrival of colder temperatures.

**FIG 3:**
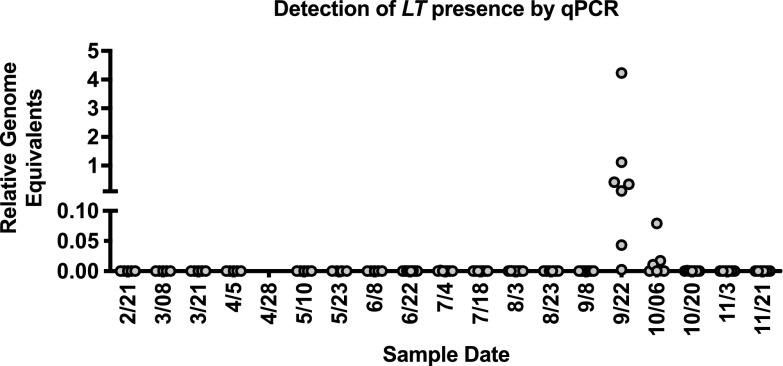
*L. thermotolerans* levels at the colony level over a single season show variable levels of colonization. *L. thermotolerans* (*LT*) cells were monitored biweekly using qPCR of pooled honey bee midguts from apiary colonies. The differences between the threshold cycle numbers for the Apis mellifera *atp5a* gene and the *L. thermotolerans* 26S rDNA gene were used to calculate the levels using the 2^−Δ^*^CT^* method (86).

For the yeast *L. thermotolerans*, we observed substantial levels in pooled midgut samples of bees for only two sampling periods (22 September and 6 October) but at no other times during the year (Fig. 3). We also observed yeast-like cells in the midgut samples by light microscopy. In order to determine whether the yeast visually identified was *L. thermotolerans*, we isolated yeast cells from pooled midguts by plating the cells onto YPD and incubating the plate overnight at 35°C (honey bee colony temperature) under aerobic conditions. We grew individual colonies in liquid culture and used fungus-specific primers NL1 and NL4 for PCR of the D1-D2 region of the large-subunit RNA for sequencing. We identified the yeast species represented in 14 isolates and found that 8/14 were *L. thermotolerans.* In addition, we observed that 2/14 were Metschnikowia pulcherrima and 2/14 were Hanseniaspora vineae, and there was 1 isolate each of Candida tropicalis and Aspergillus japonicus.

### Experimental colonization of honey bee digestive tracts by *L. thermotolerans* is temperature dependent.

**FIG 4:**
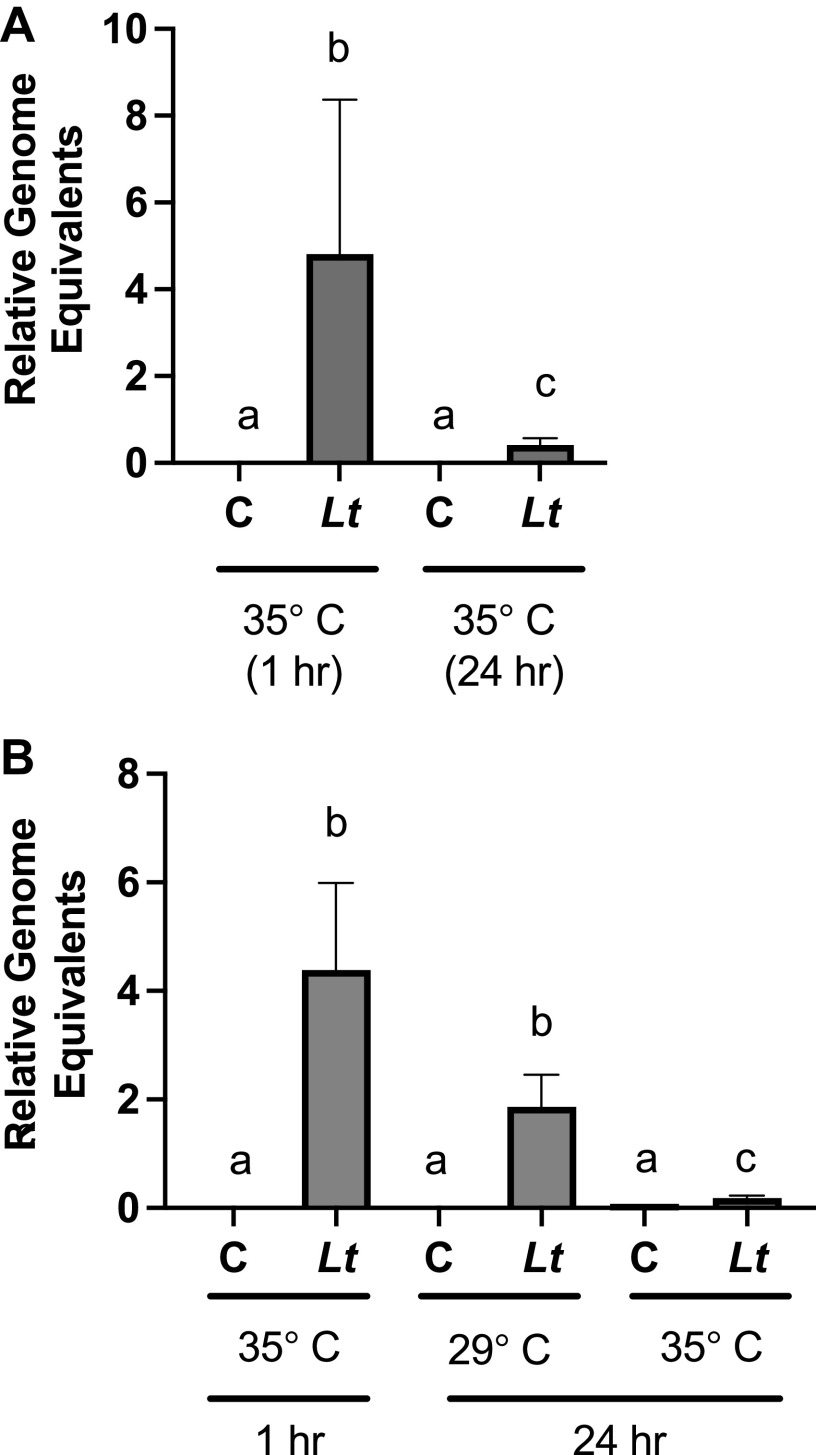
Experimental colonization of honey bee digestive tracts by *L. thermotolerans* is temperature dependent. (A) *L. thermotolerans* (*Lt*) levels were measured in bees maintained at 35°C for 1 h postinoculation and then in bees maintained at 35°C for 24 h postinoculation (C = control unexposed bees). (B) *L. thermotolerans* levels were measured in bees maintained at 35°C for 1 h postinoculation and then again in bees maintained at either 35°C or 29°C for 24 h postinoculation. The differences between the threshold cycle numbers for the Apis mellifera *atp5a* gene and the *L. thermotolerans* 26S rDNA gene were used to calculate yeast levels using the 2^−Δ^*^CT^* method (86). “A does not equal sign b does not equal sign c, *P < *0.05”, however the proof program would not allow me to insert the special symbol “do not equal sign”.

We used a soak inoculation method to introduce yeast cells cultured overnight to newly eclosed bees (31). We used qPCR to determine *L. thermotolerans* DNA levels relative to host DNA levels in a cohort of bees 1 h after inoculation to confirm the uptake of *L. thermotolerans* and another cohort 24 h after inoculation to measure colonization. We found that the soak method allowed the significant introduction of *L. thermotolerans* after 1 h. However, we observed a marked reduction in *L. thermotolerans* levels 24 h after inoculation (Fig. 4A). Based on the reported optimal growth temperature of *L. thermotolerans* of 29°C, we explored whether shifting inoculated bees to this temperature would increase colonization by *L. thermotolerans*. After 24 h, we observed that at 29°C, there were similar or even increased levels of *L. thermotolerans* relative to the levels 1 h after inoculation, while for the bees maintained at 35°C, there was again a sharp reduction in yeast levels in the midgut relative to the levels 1 h after inoculation (Fig. 4B). To confirm the growth dynamics of our isolate of *L. thermotolerans* at different temperatures *in vitro*, we grew the yeast in liquid culture overnight at 29°C, diluted the culture to an optical density (OD) at 600 nm of ~0.15, and incubated the culture at 29°C or 35°C for 24 h, with OD measurements being made at the specified times. We found that *L. thermotolerans* grown at the high temperature had a lower OD at 24 h (Fig. S4A). Staining of cells with FB28 revealed similar morphologies and sizes for yeast cells cultured at both temperatures (Fig. S4B). In addition, we found that when *L. thermotolerans* cells grown overnight were plated and grown at 29°C or 35°C for 24 h, the numbers of CFU were similar, but the final colony diameter was significantly smaller at the higher temperature (data not shown).

When we cultured the yeast cells continuously (with daily 1:100 dilutions) for more than a week at 35°C (and in parallel at 29°C). We found that yeast cells cultured in this way could still not grow to densities similar to those of yeast cells grown at 29°C despite this long exposure to the elevated temperature (Fig. S4C). Using this “heat-adapted” yeast (Lt(h)) in our experimental colonization experiments, we found that yeast cells cultured at elevated temperatures were similar to the original isolate cultured at 29°C in their temperature dependence. The Lt(h) levels were higher when bees were maintained at 29°C than when bees were kept at 35°C (Fig. S4D).

### Yeast colonization impacts the levels of Gilliamella apicola in the digestive tract of newly eclosed bees but not older bees.

**FIG 5:**
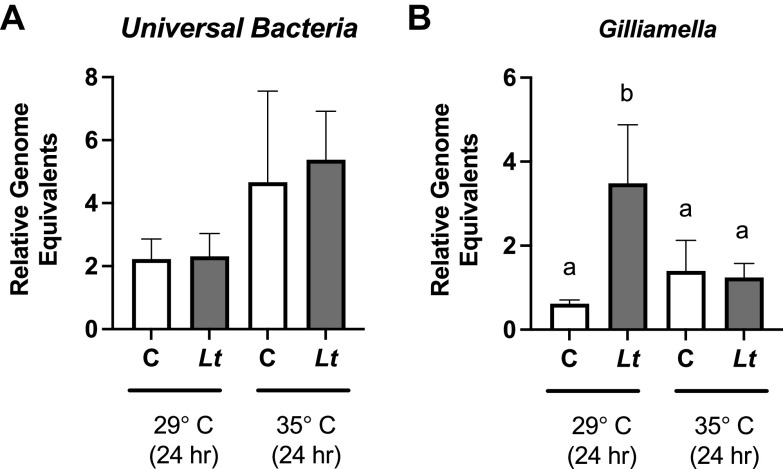
Experimental colonization of honey bee digestive tracts by *L. thermotolerans* impacts the microbiome of newly emerged bees. Levels of universal bacteria (A) and *G. apicola* (B) were measured in newly eclosed bees maintained at 35°C for 1 h postinoculation and then for 24 h postinoculation at 29°C or 35°C (C = control unexposed, Lt = *L. thermotolerans*). The differences between the threshold cycle numbers for the Apis mellifera *atp5a* gene and the bacterial 16S rRNA gene were used to calculate bacterial levels using the 2^−Δ^*^CT^* method (86). “A does not equal sign b, *P < *0.05”, however the proof program would not allow me to insert the special symbol “do not equal sign”.

To determine whether yeast colonization might lead to alterations in the microbiome, we used qPCR to measure the levels of the core bacterial species known to be part of the honey bee digestive tract microbiota (in the midgut, including the pylorus and half of the ileum) in newly eclosed bees fed yeast for 24 h as described above. We used primer sets amplifying the 16S rRNA regions of all bacteria as well as sets that amplify the species-specific 16S rRNA regions of seven bacterial species, including Gilliamella apicola, Frischella perrara, Snodgrassella alvi, Bartonella apis, Bifidobacterium asteroides, *Lactobacillus* Firmicutes species 4 (Firm-4), and *Lactobacillus* Firmicutes species 5 (Firm-5). At 24 h postinoculation, we observed no differences in the relative amounts of total bacteria under either temperature condition (Fig. 5A). In these newly eclosed bees, most bacterial subsets were highly variable and present at very low levels. However, we found that the level of G. apicola was increased after yeast feeding at 29°C for 24 h but not at 35°C (Fig. 5B).

**FIG 6:**
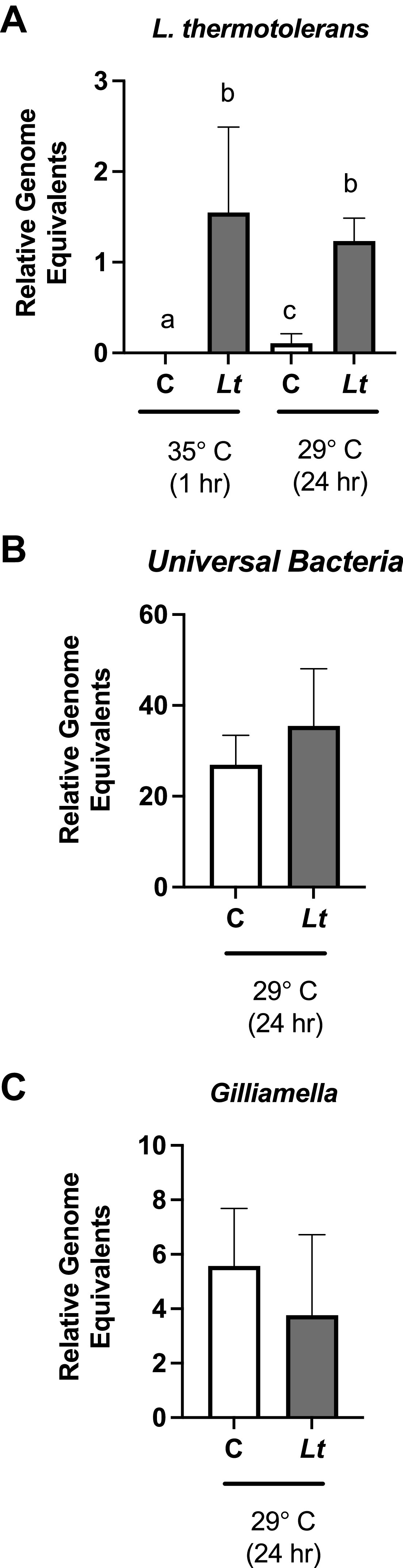
Experimental colonization of honey bee digestive tracts by *L. thermotolerans* does not impact the microbiome of older bees. (A) *L. thermotolerans* levels were measured in bees collected from the landing board of a colony and maintained at 35°C for 1 h postinoculation and then in bees maintained for 24 h postinoculation at 29°C (C = control unexposed, Lt = *L. thermotolerans*). (B and C) Levels of universal bacteria (B) and *G. apicola* (C) were measured in the landing board bees from panel A. The differences between the threshold cycle numbers for the Apis mellifera *atp5a* gene and the bacterial 16S rRNA gene were used to calculate bacterial levels using the 2^−Δ^*^CT^* method (86). “A does not equal sign b does not equal sign c, *P < *0.05”, however the proof program would not allow me to insert the special symbol “do not equal sign”.

Because newly emerged bees have not yet acquired the typical digestive tract microbial community (32, 33), we hypothesized that yeast would have less of an effect on the more established microbiome found in older bees. First, we established that we could colonize older bees taken from the landing board with *L. thermotolerans* (Fig. 6A). Next, we used the 16S rRNA primers to examine the relative levels of all bacteria as well as the levels of the specific bacterial species *G. apicola* in these landing board bees. At 24 h postinoculation, we observed no differences in the relative levels of total bacteria under either temperature condition (Fig. 6A) or in the levels of *G. apicola* (Fig. 6B).

## DISCUSSION

We describe the isolation and characterization of a yeast species found in the honey bee digestive tract in a variable manner. This represents the third known detection of *L. thermotolerans* in association with honey bees (34, 35) and the first appreciating the variable nature of its presence in honey bee midguts. *Lachancea thermotolerans* is a ubiquitous species found in diverse anthropic and wild habitats that cover a large geographic span (36). Most studies of this yeast species are focused on its potential use in viniculture, and it is a common constituent of the grape/wine microbiota (36, 37). It is noteworthy that 12 of the 14 yeast isolates from this study are yeasts associated with both the controlled fermentation of viniculture and the natural fermentation of rotting fruit. One of these yeasts, M. pulcherrima, was found to be associated with bee bread in another study (21), suggesting that many of these species may be associated with bees more commonly. It is also noteworthy that both *L. thermotolerans* and yeasts of the *Metschnikowia* genus are found in floral nectar.

The interactions among plants, pollinators, and microbes, including yeast, are known to be quite complex and likely contribute significantly to maintaining key ecological relationships (38). Madden et al. developed the “dispersal-encounter” hypothesis proposing a mutualistic relationship where insects aid in yeast dispersal across sugar resources and benefit from the presence of yeast as a signal of reliable sugar food sources (39). Previous studies in bumble bees (40–43) have found evidence that some pollinators choose flowers based in part on yeast detection. In the case of honey bees, Good et al. showed that unlike bacterial colonization, which decreased nectar consumption, yeast colonization of nectar did not decrease honey bee nectar consumption compared to nectar without yeast (44). However, evidence has also been reported that honey bees even avoid yeast-laden nectar (45). *Lachancea thermotolerans* itself, when found on the common tansy, is attractive to the mosquito pollinator of this plant (46), providing one example where the yeast described here impacts pollinator preference. Thus, from an ecological standpoint, honey bee exposure to *L. thermotolerans* might be expected to be quite common in a manner dependent on local conditions.

However, the variability in the presence of *L. thermotolerans* in the midgut is consistent with the earliest research into yeasts associated with bees (reviewed in reference 17) as well as more recent research (23). Decker et al. suggested that the fungal community in bees is not stable and pointed to the foraging location as a key determinant of the fungal species composition of the digestive tract (23). *L. thermotolerans* and yeasts of the *Metschnikowia* genus were the species most commonly detected in the bees assayed here. As these are found in floral nectar, this provides some evidence that foraging practices at the time played a role. Other factors known to influence the prevalence and abundance of fungal species are coinfections with other microbes such as V. ceranae (26), the presence of stresses such as pesticides (9, 15), life stages (25), the bacterial microbiome (47), and social status (22, 33). It is noteworthy that the presence of yeast was observed just after a round of fumagillin treatment, which may suggest that some interplay between *L. thermotolerans* and V. ceranae is involved. The impact of season has been observed for other denizens of the digestive tract of honey bees, including their bacterial microbiota (48), microsporidian species (49–51), and *Trypanosomatidae* species (52), and this may play a role in our results as well.

We detected the presence of *L. thermotolerans* in the midguts of bees only in the early fall in our limited apiary-wide analysis. More extensive sampling would be required to establish the true seasonality of the presence of *L. thermotolerans* in honey bee midguts. The variability that we observed may be due to seasonal differences in exposure to this yeast or seasonal differences in its ability to colonize the midguts of exposed bees. While there are likely many factors at play, one variable that likely plays a role in both of these aspects is temperature. *Lachancea thermotolerans* grows optimally at 29°C (53). It is possible that it can be found in the environment at high levels only as the lower temperatures of fall set in. However, another possibility is that colony temperature may influence growth. Honey bees maintain their hives at an optimal temperature of 35°C through thermoregulation. This colony-level homeostatic regulation of hive temperature is an important adaptive feature of honey bees (54–57) and is well above the optimal growth temperature of *L. thermotolerans*. Our observations in caged experiments that this yeast maintains high levels in the honey bee midgut only at temperatures below the typical colony temperature provide evidence in support of host temperature as a key determinant of *L. thermotolerans* colonization.

In our experimental colonization experiments, we do not observe an increase in yeast levels under any condition. The amount of yeast in the midgut can be considered to be influenced by the yeast input levels, the rate of yeast cell death, the rate of yeast cell proliferation, and the rate of removal (perhaps via transit to the hindgut). We have measured only the input level, and additional work quantifying the other variables would be valuable for understanding the population dynamics of the yeast in the bee digestive tract. Interestingly, in our colony-level trials, we found that some individual bees had quite high levels of yeast (with the highest observed level being 5 × 10^6^ yeast cells per bee) (Fig. 1), which may suggest that there can be very high initial inoculation levels, yeast growth is possible in some context, or both. Further work will be necessary to fully disentangle this issue. Other fungal species that colonize bees and cause disease are also influenced by colony temperature. For example, chalkbrood disease caused by the fungus *Ascosphaera apis* is most often associated with the spring season, when cooler temperatures and damp conditions provide the optimal environment for its proliferation (6, 58). Furthermore, it was recently hypothesized that the success of some of the pathogens of adult bees, including microsporidian and trypanosome species, is due to their adaptation to the higher temperatures of honey bee colonies (59–62).

For honey bee colonies, the narrow, elevated temperature range provided by endothermy is commonly thought to impart multiple advantages, such as allowing individual bees to initiate flight without warm-up, maintaining an increased rate of brood development even when the ambient temperature is low, and preserving a large colony throughout the winter, which can build up quickly in the spring (reviewed in reference 56). However, another potential advantage of high colony temperature that has been largely overlooked is pathogen resistance (56). This possibility was initially introduced by Seeley (56), who argued that once the primary factors mentioned above led to the selection of some level of nest warming, other selective pressures, including infectious disease, could continue to favor the maintenance and even the enhancement of the phenomenon. In his discussion of the potentially protective nature of elevated colony temperatures against infection, Seeley notes that this protection could extend to fungal, bacterial, and viral infectious agents. An interesting theoretical experiment comes from parallel studies in mammals. Endothermy, homeothermy, and elevated body temperatures are fundamental aspects of mammalian physiology that are also hypothesized to play a role in limiting infection by fungal species by allowing mammals to maintain a “restrictive” body temperature above the upper thermal limits of most fungal species (63, 64). Robert and Casadevall found that while 90% of fungal species are able to survive relatively well at 30°C, survival is decreased by ~6% with every 1°C increase from 30°C to 40°C (63). Considering bee nest thermoregulation as a system analogous to mammalian endothermy, we could similarly predict that ~90% of fungal species could grow in the nests of solitary bees and in the nests of other social bees with lower nest temperatures, while a honey bee colony with a temperature of 35°C would permit the growth of only ~54% of fungal species. *L. thermotolerans* is not observed in the summer months when colony thermoregulation is keeping most bees at a high temperature of 35°C, supporting the idea that colony thermoregulation limits the growth of at least this yeast species. However, the observation that *L. thermotolerans* is no longer found later in October clearly suggests that other factors are important as well. The results from our caged experiments also strongly support host temperature as being a factor in colonization by *L. thermotolerans*. The finding that *L. thermotolerans* is observed at high levels only in foragers in colonies that are in the transition between the summer mode and the winter cluster may be instructive. During this time, the brood nest at the colony core may be maintained in the optimal temperature range, although bees at the periphery, which likely are foragers, will be exposed to cooler temperatures much more amenable to *L. thermotolerans* growth.

Reduced temperature may also impact the susceptibility of the host to colonization through impacts on host function in addition to direct effects on yeast growth. While the effects of cold stress have not yet been studied in detail in adults, honey bee larvae maintained at suboptimal temperatures possess differences in physiology, behavior, and immune function (see reference 65 and references therein). Future studies to ascertain the impacts of moderate and extreme cold stress on aspects of adult bee biology will be required to understand the role of host dysfunction in *L. thermotolerans* colonization. In addition to the effects on the host cells and tissues themselves, temperature may also impact the microbiome, which could represent a key barrier to yeast colonization. For example, one honey bee symbiont, Bombella apis, was recently shown to protect against fungus-mediated disease (47). It was previously shown that newly emerged bees have minimal digestive tract bacteria and acquire the typical microbiome community only 4 to 6 days after emergence in the typical colony environment (32). Thus, the ability of *L. thermotolerans* to colonize both newly eclosed bees with an immature microbiome and older bees from the colony possessing more established bacterial communities in their digestive tracts provides some preliminary evidence that the microbiome is not dramatically impacting yeast colonization here.

A plethora of recent studies have shown that the gut microbiota of honey bees is quite complex (66) and that its composition can have a significant impact on honey bee health (67). The microbiome community provides benefits to the honey bee host, which include metabolic contributions (68) and immune modulation (69, 70). Perturbation of the honey bee microbiota by diverse mechanisms such as antibiotic exposure or dietary alterations can negatively impact honey bee health. While these studies on the microbiome have focused largely on bacterial members of the microbiota, alterations in nonbacterial gut residents, such as yeasts, may also be important contributors to honey bee health. New studies have shown yeasts to be associated with honey bees, their colonies, and their habitats (21–24). There is a growing appreciation for the potential impact of flower-associated microbes, including yeasts, on pollinators (71). Bees have been reported to consume yeast spores as a nutritional supplement during times of pollen scarcity (72), and recent evidence suggests benefits for the consuming bees (73). Similarly, colony growth benefits have been reported for bumble bees that consume nectar containing yeasts (74). Yeasts may also be important for generating or depleting certain metabolites. For example, *L. thermotolerans* is known to produce lactate very efficiently, although the importance of this molecule for honey bee health is not clear. In addition, the resident yeasts may influence other aspects of health, such as immune function and colonization by other pathogens (26, 27). It is reasonable to expect that colonization of the bee digestive tract could also impact the structure and function of the well-characterized bacterial microbiome. Here, we do not find a difference in total bacteria in the digestive tract of bees after colonization by *L. thermotolerans*. However, we observe that the consumption of yeast by newly eclosed bees can influence the level of at least one of the core members of the digestive tract microbiome, the Gram-negative organism *G. apicola* (75). The restriction of an effect to *G. apicola* is logical in light of previous reports that this bacterial species is most prominent at this stage after eclosion (76). *G. apicola*, which is actually a diverse collection of closely related subspecies (77), is thought to be an important fermenting bacterium in the gut of bees (78). This species can also metabolize sugars that are undigestible by the honey bee host (79) or, in some cases, even toxic (80, 81). It is unclear what specific changes in the digestive tract environment lead to increased *G. apicola* levels, whether this is a direct or an indirect effect of *L. thermotolerans* on *G. apicola*, and what, if any, effect this increased level might have on the health of the honey bee host (33).

Interestingly, we observed an increased level of *G. apicola* only in newly eclosed bees maintained at 29°C but not in those maintained at 35°C. It is likely that the temperature dependence of this finding is due to the reduced growth of *L. thermotolerans* at the higher temperature, although other possible explanations exist. It was previously shown that newly emerged bees have minimal digestive tract bacteria and acquire the typical microbiome community only 4 to 6 days after emergence in the typical colony environment (32). Our results with foragers also suggest that *L. thermotolerans* is not able to influence *G. apicola* levels once this stable community is established, despite high levels of colonization in older bees. While we observe yeast-induced perturbation of only a single microbiome species in a manner that is very context-dependent, there may be additional subtle differences that were simply not detected using the lower-resolution methods applied here. It is important to note that we examined the midgut (including the pylorus and half of the ileum) and therefore were not able to quantify yeast levels (or bacterial microbiome levels) in the hindgut. Another important caveat is that while the newly emerged bees used here did undergo natural eclosion and had access to a frame for a few hours, we did not pursue other bacterial inoculation methods as we were concerned that the process of introducing the core bacterial microbiome would unintentionally expose bees to fungi or V. ceranae. Thus, the bees used in this experiment did not have microbiome exposures that mimic natural colony inoculation, likely impacting their long-term microbiome (33). However, as our experiments were performed over a very short timescale (within 1.5 days of eclosion), the formation of a stable microbiome would not yet be expected, even if more robust inoculation methods had been pursued. Thus, while our results suggest that environmental microbes can impact the development of the microbiome in recently eclosed bees, further experiments to better understand how microbiome inoculation levels and timing might impact the relationship between *L. thermotolerans* and the bacterial microbiota are warranted.

We report the isolation and characterization of a yeast species in the honey bee digestive tract, subsequently identified as *L. thermotolerans*. Based on a limited apiary survey, the presence of this yeast in honey bees was detected only in the fall, perhaps suggesting a seasonal component to colonization. Oral infection experiments suggest that this yeast grows to high levels in the honey bee midgut only at temperatures below the optimal colony temperature. We also observe that *L. thermotolerans* inoculation causes microbiome perturbations in newly eclosed bees but not older bees, suggesting an interplay between this environmental yeast and the bacterial constituents of the microbiome. Thus, future studies on the relationship between environmental yeasts such as *L. thermotolerans* and honey bee health are warranted.

## MATERIALS AND METHODS

### Honey bee colonies.

Honey bees were collected from outbred colonies in New York, NY, consisting of a typical mix of Apis mellifera subspecies found in North America, at different times from February to November. Source colonies (the Barnard College typically houses 5 to 8 colonies) were visually inspected for symptoms of common bacterial, fungal, and viral diseases of honey bees.

### Yeast species identification, isolation, and characterization and apiary monitoring.

An infected midgut (verified by microscopy) was crushed in TRIzol, and RNA was extracted and converted to cDNA as described below. PCR was performed on this cDNA sample with Promega (Madison, WI) GoTaq PCR reagents using fungal primers. The primers ITS1F and ITS2 were used to amplify the ITS1 region of the fungal rRNA operon (82). NL1 and NL4 (83) were then used to amplify the D1-D2 region of the large-subunit RNA to confirm species identification. In all cases, PCR products were then purified using a QIAquick PCR purification kit and TA cloned, and plasmids from individual clones were subsequently sent for sequencing (Genewiz, NJ). The resulting sequences were then run through a BLAST nucleotide search (NCBI).

For yeast isolation, an aliquot of the infected midgut described above was plated onto yeast extract-peptone-dextrose (YPD) containing streptomycin (50 μg/mL) to prevent bacterial growth. The plates were incubated at 34°C (honey bee colony temperature) for 24 h under aerobic conditions. A single colony was isolated, and further growth of the yeast species was subsequently performed using YPD agar or YPD broth. For species confirmation and qPCR primer validation, DNA and RNA were extracted from aliquots of clonal yeast liquid cultures, and cDNA was prepared from RNA as described below. For growth dynamics experiments in liquid culture, yeast cells grown overnight at 29°C were diluted to an OD at 600 nm of ~0.15 and incubated at 29°C or 35°C for 24 h, with OD measurements being made at the times specified in the figure legends.

We used qPCR to quantify *L. thermotolerans* and V. ceranae over a single season (21 February to 21 November 2017) in our apiary (the number of colony samples at each time point can be found in Fig. S3 in the supplemental material). We used 12 bees captured at the landing board to perform our sampling by homogenizing the pooled midguts in 6 mL H_2_O using a Dounce homogenizer, followed by DNA extraction and qPCR as described below.

### Experimental colonization of honey bees by *L. thermotolerans*.

Yeast cells were cultured overnight (at 29°C unless otherwise stated) and resuspended in YPD broth at 1 × 10^8^ yeast cells/mL after counting with a hemocytometer and a light microscope. The cells were adjusted to 5.0 × 10^6^ yeast cells/mL in a sucrose solution. Newly emerged bees were collected after hatching from a capped brood frame overnight in an incubator at 35°C in the presence of PseudoQueen (Contech, Victoria, BC, Canada) as a source of queen mandibular pheromone (QMP). Approximately 20 mL of newborn honey bees was collected and placed by hand into two 50-mL conical tubes connected at the opening and sealed together by parafilm, which are called “double tubes” here. For soak inoculation of yeasts (previously pioneered by another group to deliver microbiome bacterial species to bees [31]), 1 mL of the yeast-sucrose solution was added to the tubes via a hole in one end, and parafilm was used to reseal the opening. This procedure was repeated for a second double tube that received the sucrose solution without yeast cells. Each double-tube structure was slowly tilted 180° side to side for solution distribution. To ensure oxygen exchange, a metal thumbtack was used to pierce a small hole through the parafilm on the bottom of each end of the double-tube structure. The double tubes were placed into an incubator at 35°C for 1 h to allow time for bees to consume the solution. Subsequently, each inoculated double tube was gently shaken until the honey bees were split evenly on the two sides, and the structure was bent at the openings of the tubes to rip the parafilm and empty each side into a separate cage. Approximately 20 to 30 newly eclosed bees were placed into each 12.2-cm by 8.6-cm by 21.3-cm acrylic cage with a sliding door machined at Carleton Labs, Columbia University. An InsectaVac aspirator (BioQuip, Rancho Dominguez, CA) was used to remove 10 to 12 bees from each of the cages for the assessment of yeast cell uptake. The remaining caged bees were maintained in incubators at 35°C or 29°C. For experiments with bees directly from the colony, bees were collected from the landing board and infected as described above. Honey bee colonization experiments using newly emerged bees were repeated in 8 independent trials, and colonization experiments using landing board bees were repeated in 4 independent trials.

### DNA isolation.

For sample preparation, honey bee midguts (including the pylorus and half of the ileum) were dissected and crushed in 0.5 mL water. DNA extraction was performed for individual midguts using a modified smash-and-grab DNA miniprep protocol (84). Samples were lysed by vortexing for 45 s in the presence of 0.3 mL of 1-mm glass beads in 0.2 mL of lysis buffer (10 mM Tris [pH 8.0], 1 mM EDTA, 100 mM NaCl, 1% SDS, 2% Triton X-100) and 0.2 mL of a 1:1 mix of phenol and chloroform. After the addition of 0.2 mL of Tris-EDTA (TE), mixing, and centrifugation, the aqueous layer was transferred to a clean tube, and 3 volumes of 100% ethanol were added to precipitate the DNA (1 h at −20°C). The DNA was washed with 70% ethanol, air dried, and resuspended in H_2_O.

### qPCR for *L. thermotolerans*, bacteria, and V. ceranae quantification.

qPCR was used to determine the relative levels of *L. thermotolerans*, bacterial, and V. ceranae genome equivalents versus host genome equivalents using DNA samples derived from individual honey bee midguts. For qPCR, 1 μL of DNA was then used as a template to determine the levels of various microbial groups using primers specific for the *L. thermotolerans* 26S rDNA gene (forward [F] primer 5′-CGCTCCTTGTGGGTGGGGAT-3′ and reverse [R] primer 5′-CTGGGCTATAACGCTTCTCC-3′) (30), the V. ceranae β-actin gene (F primer 5′-TCTGGTGATGGTGTCTCCCA-3′ and R primer 5′-TGCCCATCAGGCATTTCGTA-3′), or the 16S rRNA genes of various bacterial groups (85) relative to the level of the honey bee nuclear ATPase 5a gene (*atp5a*) (forward primer 5′-TCCTTACGTTTGGTTTCTTCG-3′ and reverse primer 5′-GGATCCGTATGATTATTGCAAAG-3′) (28) using iQ SYBR green supermix (Bio-Rad, Hercules, CA) in an iCycler thermocycler (Bio-Rad, Hercules, CA). The PCR conditions were as follows: 94°C for 2 min followed by 94°C for 15 s, 60°C for 30 s, and 72°C for 60 s for 40 cycles. These steps were followed by a 10-min extension step at 72°C. The differences among the threshold cycle (*C_T_*) numbers for the *L. thermotolerans* 26S rDNA gene, the V. ceranae β-actin gene, the various bacterial 16S rRNA genes, and the honey bee nuclear *atp5a* gene were used to calculate the levels of yeast infection using the 2^−Δ^*^CT^* method (86).

### Chitin staining.

For chitin staining, midguts were crushed in 0.5 mL MilliQ H_2_O per bee. After being adjusted to 10 mM Tris-HCl (pH 9), the sample was incubated with 0.001% fluorescent brightener 28 (FB28), which is also known as calcofluor white M2R (Sigma, St. Louis, MO), for 30 min at 37°C. Visualization of FB28-bound spores was performed using a Nikon (Melville, NY) Eclipse E600FN microscope at a ×40 magnification.

### Statistical analysis.

Data are presented as values from individual bees, with means ± standard errors of the means (SEM) also shown. Data were compared using unpaired *t* tests with Welch’s correction when values fit normal distributions or Mann-Whitney U nonparametric tests when they did not fit normal distributions. Normality was assessed using Shapiro-Wilk tests. When more than two groups were being compared, data were analyzed using one-way analysis of variance (ANOVA) with Dunnett’s T3 multiple-comparison test when values fit normal distributions or a Kruskal-Wallis test when they did not. For correlations, Pearson’s correlation analysis was used for comparing normally distributed populations, and Spearman’s correlation analysis was used for comparing nonnormally distributed populations.
