# Supplementary material for: Colonization of Honey Bee Digestive Tracts by Environmental Yeast Lachancea thermotolerans Is Naturally Occurring, Temperature Dependent, and Impacts the Microbiome of Newly Emerged Bees
Source: Microbiol Spectr. 2023 Feb 15;11(2):e05194-22. doi: 10.1128/spectrum.05194-22 (PMC10100982; doi:10.1128/spectrum.05194-22)
Supplement: Supplemental file 1 — Fig. S1 to S4. Download spectrum.05194-22-s0001.pdf, PDF file, 11.9 MB [file spectrum.05194-22-s0001.pdf]

Supplementary material

Supplementary Figure 1. Midgut preparations from a *V. ceranae*-infected bee and a yeast-infected bee with or without Fluorescent Brightener 28 were visualized using UV using a 40x objective.

Supplementary Figure 2. Sequence from the RNA extracted from a liquid culture inoculated with a single colony of the plated yeast using fungal-specific primers for the ITS1 region of the fungal rRNA operon or D1-D2 region of the large-subunit RNA for sample.

Supplementary Figure 3. Number of colonies sampled per timepoint during *L. thermotolerans* and *V. ceranae* monitoring (A). *V. ceranae* infection levels over a single season during biweekly monitoring (B). For qPCR, the difference between the threshold cycle number for *Apis mellifera Atp5a* and a gene of the microbe of interest (*V. ceranae β-actin* gene or *L. thermotolerans 26S rDNA* gene) was used to calculate the level of infection using the $2^{(-\Delta CT)}$ method (Schmittgen and Livak 2008). $*P < .05$; $**P < .01$

Supplementary Figure 4. OD600 values obtained at the specified times (0, 6, and 24 hours) for *L. thermotolerans* incubated at 29 °C or 35 °C for 24 hours (A). Morphology of *L. thermotolerans* incubated at 29 °C or 35 °C for 24 hours showed via staining with FB28 and visualized using UV with 40x objective (B). *L. thermotolerans* were incubated continuously at 29 °C or 35 °C for 7 days (with 1:100 dilutions each day). OD600 values for each strain at 0 and 24 hours on day 7 at their respective temperatures (C). Levels of the heat adapted *L. thermotolerans* (LTe) were

measured in bees maintained at 35 °C for 1 hour post-inoculation and then again at for bees maintained at either at 35 °C or at 29 °C 24 hours post-inoculation (D).

Kogan_Suppl.Fig1.

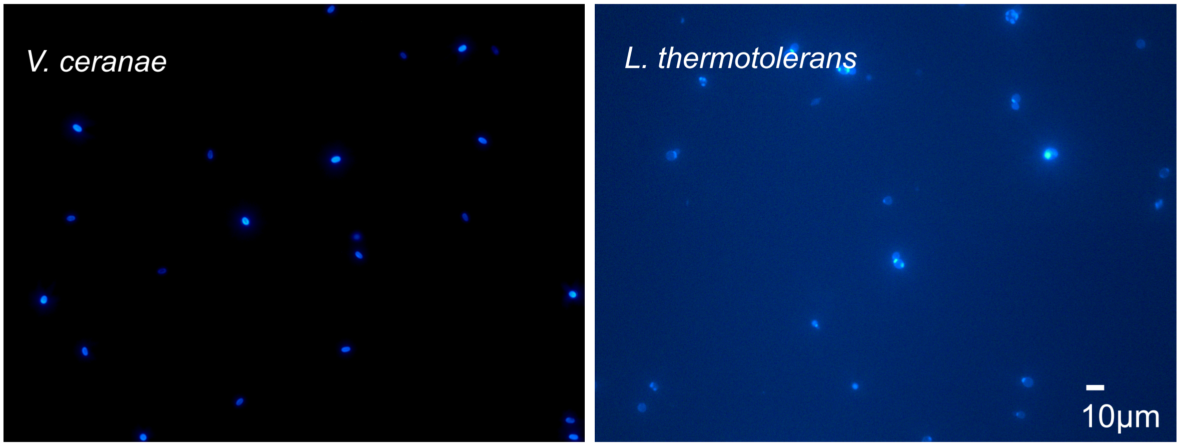

Supplemental Figure 2

> ITS1 region of the fungal rRNA operon for sample
CTTGGTCATTTAGAGGAAGTAAAAGTCGTAACAAGGTTTCCGTAGGTGAACCTGCGG
AAGGATCATTAAAGAATTTTGTTAGAGCAGCCGGGAAAGTTCAGGAGCCTGCGCTT
GGTTGCGCGGCCGATGATGCTTTCTGTTAACGACTGTCTCTCTACACACACACTGTG
GAGTAATTTATTTTACAACGCTTCTTCTTTGGGCTTTACGGCCCAAGGGTTACAAACA
CAAACAACTATTGTATTTTAAACATTGTCAATTATTTTTCATTTTAGAAAAAAAATAT
TTAAAACTTTCAACAACGGATCTCTTGGTTCTCGCATCGATGAAGAACGCAGC

> D1-D2 region of the large-subunit RNA for sample
GGTCCGTGTTTCAAGACGGGCGGCATTTAACCATTATGCCAACATCCTTGACAAAAG
TCGCAGTCCTCAGTCCCGGCTGGCAGTATTCCTCTGGGCTATAACGCTTCTCCGAAG
ACAAGCCACGTTCCCAAAGATTTATCCTACCGCCAAAACTGATGTTGGCCCAGTGAG
CTGCGAGATCCCCACCCACAAGGAGCGAGGGTCGCAAAACACCATGTCTGATCAAA
TGCCCTTCCCTTTCAACAATTTCACGTACTTTTTCACTCTCTTTTCAAAGTTCTTTTCA
TCTTTCCATCACTGTACTTGTTCGCTATCGGTCTCTCGCCAATATTTAGCTTTAGATG
GAATTTACCACCCACTTAGAGCTGCATTCCCAAACAACTCGACTCGTCGAAAGCACT
TTACATAGGACTAGACTCCTCGCCATACGGGATTCTCACCCTCCATGACGTCCTGTT
CCAAGGAACATAGACAAGGACTAGCCCCAAAGTAGCTTCTTCAAATTACAACTCGG
ACACCGAAGGTGCCAGGTTTCAAATTTGAGCTTTTGCCGCTTCACTCGCCGTTACTA
AGGCAATCCCGGTTGGTTTCTTTTCCTCCGCTTATTGATATGC

Kogan_Suppl.Fig3.

A

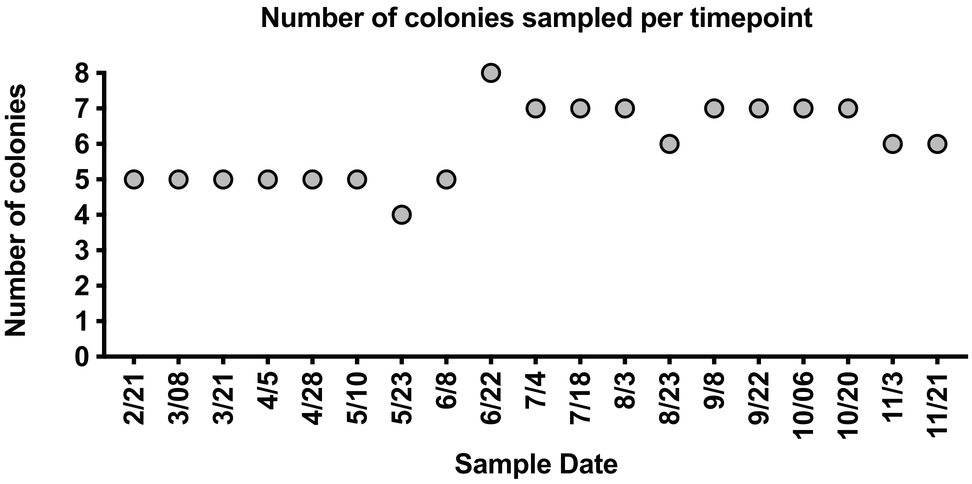

B

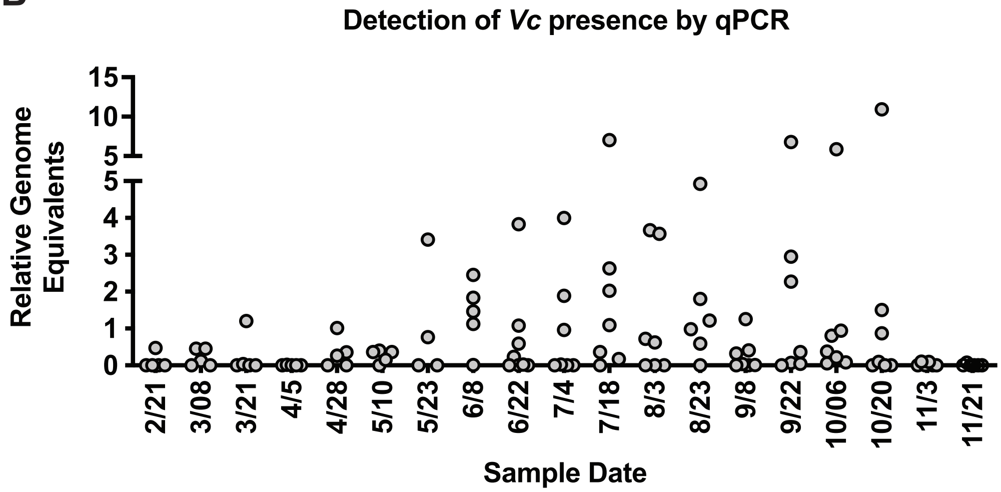

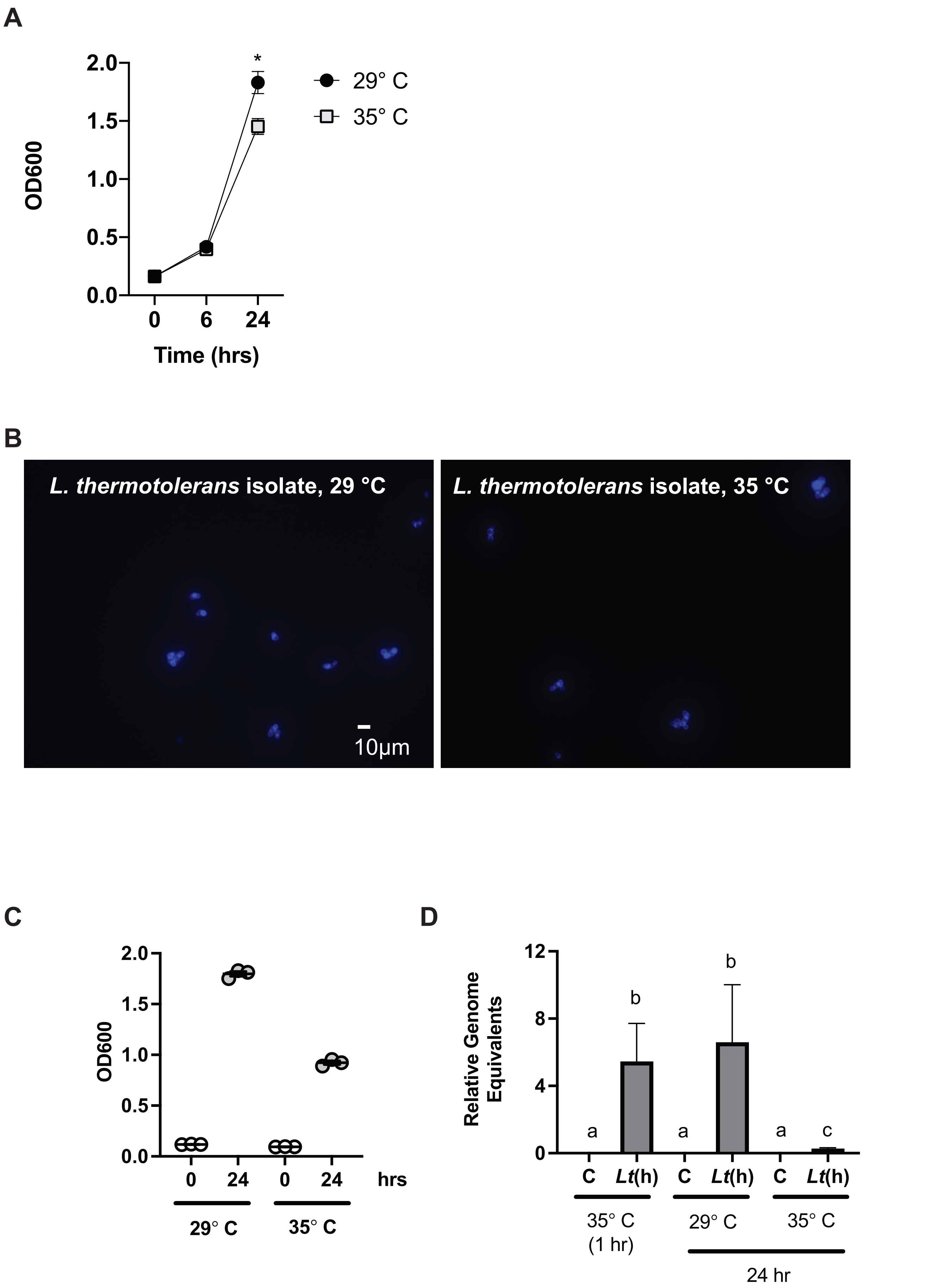
A
2.0
1.5
1.0
0.5
0.0
OD600
*
29° C
35° C
0
6
24
Time (hrs)
B
L. thermotolerans isolate, 29 °C
10µm
L. thermotolerans isolate, 35 °C
C
2.0
1.5
1.0
0.5
0.0
OD600
0
24
0
24
hrs
29° C
35° C
D
12
8
4
0
Relative Genome Equivalents
a
b
a
b
a
c
C
Lt(h)
C
Lt(h)
C
Lt(h)
35° C (1 hr)
29° C
35° C
24 hr
